# Stress and psychological impact of the COVID-19 outbreak on the healthcare staff at the fever clinic of a tertiary general hospital in Beijing: a cross-sectional study

**DOI:** 10.1192/bjo.2021.32

**Published:** 2021-04-05

**Authors:** Xia Hong, Jinya Cao, Jing Wei, Yanping Duan, Xiaohui Zhao, Jing Jiang, Yinan Jiang, Wenqi Geng, Huadong Zhu

**Affiliations:** Department of Psychological Medicine, Peking Union Medical College Hospital, Chinese Academy of Medical Sciences & Peking Union Medical College, China; Department of Psychological Medicine, Peking Union Medical College Hospital, Chinese Academy of Medical Sciences & Peking Union Medical College, China; Department of Psychological Medicine, Peking Union Medical College Hospital, Chinese Academy of Medical Sciences & Peking Union Medical College, China; Department of Psychological Medicine, Peking Union Medical College Hospital, Chinese Academy of Medical Sciences & Peking Union Medical College, China; Department of Psychological Medicine, Peking Union Medical College Hospital, Chinese Academy of Medical Sciences & Peking Union Medical College, China; Department of Psychological Medicine, Peking Union Medical College Hospital, Chinese Academy of Medical Sciences & Peking Union Medical College, China; Department of Psychological Medicine, Peking Union Medical College Hospital, Chinese Academy of Medical Sciences & Peking Union Medical College, China; Department of Psychological Medicine, Peking Union Medical College Hospital, Chinese Academy of Medical Sciences & Peking Union Medical College, China; Department of Emergency, Peking Union Medical College Hospital, Chinese Academy of Medical Sciences & Peking Union Medical College, China

**Keywords:** Psychosocial interventions, Impact of Event Scale, Stress, COVID-19, frontline healthcare workers

## Abstract

**Background:**

It is important to maintain the psychological well-being of front-line healthcare staff during the coronavirus disease 2019 (COVID-19) pandemic.

**Aims:**

To examine COVID-19-related stress and its immediate psychological impact on healthcare staff.

**Method:**

All healthcare staff working in the fever clinic, from 20 January 2020 to 26 March 2020, of a tertiary general hospital were enrolled. Stress management procedures were in place to alleviate concerns about the respondents’ own health and the health of their families, to help them adjust their work and to provide psychological support via a hotline. Qualitative interviews were undertaken and the Sources of Distress and the Impact of Event Scale-Revised (IES-R) were administered.

**Results:**

Among the 102 participants (25 males; median age 30 years, interquartile range (IQR) = 27–36), the median IES-R total score was 3 (IQR = 0–8), and 6 participants (6.0%) scored above the cut-off on the IES-R (≥20). Safety and security were acceptable or better for 92 (90.2%) participants. The top four sources of distress were worry about the health of one's family/others at 0.88 (IQR = 0.25–1.25), worry about the virus spread at 0.50 (IQR = 0.00–1.00), worry about changes in work at 0.50 (IQR = 0.00–1.00) and worry about one's own health at 0.25 (IQR = 0.25–0.75). There was a moderate correlation between the IES-R score and the Sources of Distress score (rho = 0.501, *P* = 0.001).

**Conclusions:**

The stress levels of healthcare staff in the fever clinic during the COVID-19 epidemic were not elevated. Physio-psychosocial interventions, including fulfilment of basic needs, activation of self-efficacy and psychological support, are helpful and worth recommending in fighting COVID-19.

## Background

Coronavirus disease 2019 (COVID-19) has become a worldwide pandemic. The severe acute respiratory syndrome (SARS) epidemic in 2003 was controlled through numerous measures in China, including the establishment of fever clinics for triaging patients.^1^ COVID-19 was placed at the highest alert level throughout China on 20 January 2020. A special 24 h fever clinic was set up to triage patients and fight COVID-19 in the Department of Emergency, Peking Union Medical College Hospital on the same day.^2^ Because healthcare personnel work with infected patients at the front line in emergency situations in unpredictable and possibly life-threatening circumstances, their stress came not only from routine work but also from COVID-19-related psychological stress.

## Lessons from SARS

During the outbreak of SARS, a high degree of distress, indicated by a high score on the Impact of Event Scale (IES),^3^ was experienced by 17.7–36.0% of hospital workers.^4–7^ SARS caused a significant level of distress among emergency department staff, and the most important variables that could account for the distress levels were loss of control/vulnerability, fears about one's own health and spread of the virus among emergency department staff.^8^ Based on the experiences and lessons from responding to SARS, procedures to manage these sources of stress may decrease post-traumatic stress and maintain front-line healthcare staffs’ well-being when fighting COVID-19.

## Aims

The objectives of the present study were to examine COVID-19-related stress and its immediate psychological impact among healthcare staff in the fever clinic, to help improve the management of the stress of healthcare staff and maintain their psychological well-being during a pandemic of acute contagious disease.

## Method

### Participants

Thirty-seven healthcare staff in a first group (group 1) and 68 healthcare staff in a second group (group 2) stayed and worked in the hospital for 2–3 weeks and then left the fever clinic. The selection criteria for assigning healthcare staff to the fever clinic included:

(a) having at least 2 years’ experience of clinical work;
(b) being recommended by various departments on a voluntary basis;
(c) having good communication skills and the ability to cooperate;
(d) having received careful training on infectious disease prevention and related knowledge before taking up the post.

On completion of their assignments, the staff then quarantined and convalesced in a vocational resort for 2 weeks. During their rotation in the fever clinic, a separate apartment building with an individual bedroom in the hospital was offered to each of healthcare staff, and stays in the building after work were required. The date of departure from the fever clinic was from 3 February to 17 February for group 1 of the staff and from 20 February to 26 March for group 2 of the staff. Qualitative and quantitative evaluations via telephone were conducted by trained psychiatrists and psychological evaluators 1 to 5 days after their departure from the fever clinic. The interviews were conducted from 6 February to 19 February for group 1 and from 25 February to 28 March for group 2.

The study was approved by the ethics committee of Peking Union Medical College Hospital (approval number: S-K1045). Oral informed consent was obtained before the interviews began. All healthcare staff at the fever clinic during that time were eligible for the study; 105 healthcare staff were enrolled, 102 (97.1%) people agreed to participate and finished the interview, and 3 (2.9%) refused.

### Stress management procedures

In order to alleviate worries about the health of their families, the healthcare staff in the fever clinic stayed in the hospital during their rotation and quarantined and convalesced for 2 weeks after they left the fever clinic so that they would not infect their family members with the virus. The labour union was enrolled in the support group, which included hospital leaders, and arranged for dietary needs and accommodation and provided necessary help for healthcare staff and their family members,such as providing the family members with necessary help when they needed to see a doctor.^2^

Healthcare staff were provided with protective devices, were always supervised when they were putting on their protective equipment to make sure they put it on properly and were given standardised protection processes and training before their rotation in the fever clinic to ensure their health was protected.^9^

Reasonable adjustments to the working hours of front-line healthcare staff were made according to their feedback,^2^ and training, inspection and supervision were provided in the work environment to help medical staff adapt to the work processes as quickly as possible, and alleviate their stress at work.^9^

Since November 2011, a psychological hotline service has been available 4 h a week for healthcare staff in the hospital provided by the Department of Psychological Medicine. Experienced psychiatrists and psychological evaluators work on the hotline after receiving standardised training. The psychological hotline service was available to front-line healthcare staff in the fever clinic 7 days a week from 9:00 hours until 21:00 hours beginning on 24 January 2020, provided by the same team. The hotline workers offered human-centred psychological support including active listening, acceptance, understanding, empathy, clarification, cooperation, feedback and resource-oriented psychological support to help them explore their internal and external resources, realistic and psychological resources.

Staff working on the hotline were required to abide by the principles of confidentiality, and not discuss any information related to work on the hotline on other occasions, except for reporting their work to the hotline supervisor. Staff working on the hotline were required to treat information held by them about the front-line staff anonymously when they reported on their work to the hotline supervisor, as well as when the hotline supervisor provided feedback to leaders of the treatment team. Exceptions were circumstances when a hotline worker and their supervisor thought that the status of a front-line staff member was affecting their ability to work and their position need to be adjusted, but this did not happen in this study.

### Interview process

The research interviews were conducted by the hotline staff. In order to increase the response rate to the interview, all the surveys were conducted after healthcare staff finished their rotation in the fever clinic, so as to ensure their accessibility and that they had time to be interviewed. The process was for healthcare staff to call the hotline to complete the interview. The hotline staff contacted participants who did not call them until each person had either made an appointment to complete the interview or had refused to complete it.

Fixed guiding words to explain the purpose of the interview. Open qualitative interviews were conducted in the first step, followed by quantitative questionnaires. Hotline workers stopped the interview if necessary, to provide essential support. All interviews, whether initiated by hotline workers or participants, were conducted by telephone at the participants’ convenience.

### Measures

#### Interview

Semi-structured qualitative interviews were conducted using the following questions.

(a) How many hours have you worked in the fever clinic?
(b) How long do you think it is suitable for you to be at work per day?
(c) How is your work intensity?
(d) What kind of adjustments would make you feel better in your work?
(e) What do you think about your medical security?
(f) What aspects make you feel safe, and what aspects make you feel unsafe?
(g) How are the arrangements for your diet and accommodation? Could there be any improvements? Or have there been oversupplies of food or other goods?
(h) Do you have any other suggestions?^10^

The administrator of the hotline provided continuous feedback on findings to the Department of Emergency, the Medical Affairs Office and the labour union to allow for suitable adjustments. The individual information of the front-line staff was treated anonymously in the feedback.

The interview records were coded by scenario analysis and topics combination. Researchers read the interview records repeatedly, marked them to form the most basic open code before they integrated and summarised the relational meaning unit to gradually form the theme. After repeated reading and understanding, they compared, reflected on, deleted, split or merged the topics, so as to better achieve internal homogeneity and external heterogeneity, and to check and confirm the meaning of each topic. Some of the results have been published elsewhere.^2,10^

On the basis of this research, we extracted and converted some qualitative data into binary variables. The answers to questions (a) and (b) were converted to binary variables if the participants felt that the length of time spent at work was excessive. The answers to questions (c) and (d) were converted to binary variables if they felt that their work intensity was very high. The answer to question (e) was converted into binary variables to describe the subjective feeling of security as not good enough, acceptable/good/or very good. For answers to question (f), we counted any safe or unsafe aspects the participants mentioned spontaneously. The answer to question (g) was converted to binary variables depending whether the participants were satisfied with the arrangements for diet and accommodation.

The quantitative interviews also included demographic questions and the questionnaires mentioned below.

The sociodemographic data that were collected included age, gender, marital status, duties in the fever clinic, years spent working in the hospital, years of education and whether participants came into contact with COVID-19 patients or their specimens in their work.

#### Impact of Event Scale-Revised

The IES-Revised (IES-R) is a 22-item self-report questionnaire designed to assess symptoms of intrusive thoughts (8 items), avoidance (8 items) and hyperarousal (6 items) resulting from traumatic life events. The specific stressful life event in the current study was work at the fever clinic during the COVID-19 pandemic. The scale measures the severity with which each of the symptoms had occurred over the past 2 weeks; each item is rated on a 5-point scale using anchors between 0 (not at all) and 4 (extremely), and total scores range from 0 to 88.^11^ Good reliability and validity of the English and Chinese versions have been previously reported.^11–13^

A total score of 20 or more was interpreted as the cut-off point in the current study, as suggested by previous studies of populations affected by traumatic events – to indicate a high level of subjective stress symptoms.^7,14,15^

#### Sources of distress

Sources of distress were measured by an 18-item questionnaire.^8^ Wong et al designed the scale during the SARS outbreak in Hong Kong. The participants were asked to rate how well each item described their present situation according to a four-point Likert scale (0,  does not completely describe my situation; 3, does completely describe my situation).

The questionnaire included items that could be grouped under six subscales as follows: health of self, health of family/others, virus spread, vulnerability/loss of control, change in work and isolation.^8^ The score of each subscale equals the average of the included items. The total score of the scale is the average of the 18 items. Scores on the scale and subscales ranged from 0 to 3. The English version of the scale was translated into Chinese through translation and back translation in the current study. The internal consistency reliability (Cronbach's alpha) for the scale was 0.886, and for the six subscales was 0.735, 0.745, 0.483, 0.690, 0.598 and 0.367, respectively.

#### General Self-Efficacy Scale

The General Self-Efficacy Scale (GSES) is a 10-item self-administered scale, which assessed the strength of an individual's belief in his or her own ability to respond to novel or difficult situations and to deal with any associated obstacles or setbacks*,* such as ‘I am confident that I could deal efficiently with unexpected events’.^16,17^ For each item there are four response choices from ‘not at all true’ that scores 1 to ‘exactly true’ that scores 4.^16,17^

The scores for each of the ten items are summed to give a total score. The score on this scale reflects the strength of an individual's generalised self-efficacy belief, the higher the score, the greater is the individual's generalised sense of self-efficacy. The Chinese version of the GSES was used in this study, the scale's Cronbach's alpha is 0.87, the split-half reliability coefficient is 0.82 and test–retest reliability coefficient 0.83.^16,17^

### Statistical analysis

The Kolmogorov–Smirnov test was performed to verify whether the continuous variables fit a normal distribution. The GSES score fit to normal distribution was expressed as the mean (s.d.), and group comparisons were performed with independent-sample *t*-tests. Other continuous variables in the study that did not fit a normal distribution were expressed as the median (interquartile range, IQR), and group comparisons were performed with the non-parametric Mann–Whitney *U*-test. For categorical variables, expressed as *n* (%), χ^2^-tests were used for group comparison. Non-parametric Spearman correlation was performed to analyse the correlation between continuous variables. The significance level was set at α = 0.05, and all tests were two-tailed. Statistical analyses were performed with IBM SPSS Statistics 22.0.

## Results

### Sociodemographic data

In total 102 healthcare staff were enrolled, including 40 (39.2%) doctors, 54 (52.9%) nurses and 8 (7.8%) laboratory technicians handling specimens from patients. A total of 25 (24.5%) were men, and 46 (45.1%) were married. The participants had a median age of 30 (IQR = 27–36) years, a median of 6 (IQR = 3–13) years of work experience, and a median of 17 (IQR = 16–20) years of education. Of the participants, 93 (91.2%) had contact with patients with COVID-19 or their specimens in their work. There was no nosocomial COVID-19 infection among healthcare staff and in-patients in the hospital until 31 March 2020.

### IES-R scores

Two participants did not finish the IES-R questionnaire. The IES-R score was generally low in participants, with a total median score of 3 (IQR = 0–8), with scores of 1 (IQR = 0–4) for intrusive thoughts, 0 (IQR = 0–2) for avoidance and 1 (IQR = 0–2) for hyperarousal. There were six participants (6.0%), with 3 (3/35, 8.6%) from the first group and three (3/65, 4.6%) from the second group who reported scores on the IES-R that were above the cut-off score (≥20). In these six participants, intrusive thoughts (median 15, IQR = 13–23) was more evident than avoidance (median 9, IQR = 5–18) and hyperarousal (median 5, IQR = 4–14).

### Fulfilment of physiological/safety needs and IES-R scores

According to data extracted from qualitative interviews, 23 (62.2%) individuals in group 1 responded that the length of time spent at work was excessive at the beginning; this figure decreased to eight (21.6%) individuals after adjustments were made in working hours and shift patterns. For work intensity, 24 (64.9%) and 33 (50.8%) of the healthcare staff in group 1 and group 2, respectively, felt that their intensity was sometimes very high (Table 1).

**Table 1:** Fulfilment of physiological and safe needs

	Group 1 (*n* = 37)	Group 2 (*n* = 65)	Total (*n* = 102)	IES-R median (IQR)	*P*^a^
Work time before adjustment, %					
Excessive	23 (62.2)	–	–	7 (2–10)	0.120
Acceptable	14 (37.8)	–	–	2 (0–4)	
Work time after adjustment, %					
Excessive	8 (21.6)	9 (13.8)	17 (16.7)	7 (2–12)	0.057
Acceptable	29 (78.4)	56 (86.2)	85 (83.3)	3 (0–8)	
Work intensity, %					
Very high sometimes	24 (64.9)	33 (50.8)	57 (55.9)	4 (1–9)	0.270
Acceptable	13 (35.1)	32 (49.2)	45 (44.1)	3 (0–7)	
Security, %					
Not good enough	6 (16.2)	4 (6.2)	10 (9.8)	9 (6–16)	0.016
Acceptable, good or very good	31 (83.8)	61 (93.8)	92 (90.2)	3 (0–7)	
Satisfied with diet, %					
Yes	35 (94.6)	64 (98.5)	99 (97.1)	3 (0–8)	0.847
No	2 (5.4)	1 (1.5)	3 (2.9)	5 (4–6)	
Satisfied with accommodation, %					
Yes	37 (100)	64 (98.5)	101 (99.0)	3 (0–8)	0.911
No	0 (0)	1 (1.5)	1 (1.0)	2	
What makes one feel safe, %					
Enough protective devices	18 (48.6)	35 (53.8)	53 (52.0)	–	–
Supervision of protection procedure	10 (27.0)	27 (41.5)	37 (36.3)	–	–
Standardised protection process	8 (21.6)	12 (18.5)	20 (19.6)	–	–
On-the-job training	1 (2.7)	8 (12.3)	9 (8.8)	–	–

IQR, interquartile range.

a. *P*-value for non-parametric Mann–Whitney *U*-test of Impact of Event Scale-Revised (IES-R) scores in different groups.

In total, 92 (90.2%) participants reported their perceptions that the health security within which they worked was acceptable, good or very good compared with 10 people whose opinions were that the security was not good enough. These groups scored differently on the IES-R scale with the latter group having higher scores (*P* = 0.016).

The top five facilities or provisions mentioned spontaneously by healthcare staff as making them feel safe were: having sufficient protective devices including masks, isolation gowns, goggles and protective screens (*n* = 53, 52.0%); supervision of protection procedures (*n* = 37, 36.3%); standardised protection processes (*n* = 20, 19.6%); on-the-job training (*n* = 9, 8.8%); and disinfection measures (*n* = 9, 8.8%). Other feedback included less frequent exposure to COVID-19 patients (*n* = 3, 2.9%) and quarantine after completion of the fever clinic rotation (*n* = 1, 1.0%).

Most of the respondents were satisfied with the dietary arrangements (*n* = 99 participants, 97.0%) and their accommodation (*n* = 101 participants, 99.0%) (Table 1). Fourteen of our participants (13.7%) indicated that shortage of disposable isolation gowns made them feel unsafe.

### Sociodemographic data, IES-R and GSES scores

The average score on GSES were 29.5 (s.d. = 5.4), 29.5 (s.d. = 5.0) in group 1 and 29.0 (s.d. = 5.4) in group 2. No significant relationship existed between the IES-R and GSES (Spearman's rank correlation coefficient rho = 0.190, *P* = 0.126). The relationship between the IES-R, GSES and sociodemographic data in the form of Spearman's rank correlation coefficient rho are shown in Table 2.

**Table 2:** The Impact of Event Scale-Revised (IES-R) scores and the General Self-Efficacy Scale (GSES) scores of medical workers in the fever clinic and their correlations with sociodemographic data

	Participants, *n* = 102	IES-R, median (IQR) (*n* = 100)	Spearman correlation^a^	*P*	GSES, mean (s.d.) (*n* = 100)	Spearman correlation^b^	*P*
Age, years: median (IQR)	30 (27–36)	–	0.192	0.056^c^	–	0.042	0.673^c^
Years of education, median. (IQR)	17 (16–20)	–	–0.077	0.448^c^	–	0.003	0.979^c^
Years of work experience, median (IQR)	6 (3–13)	–	0.193	0.054^c^	–	0.029	0.772^c^
Group, *n* (%)							
Group 1	37 (36.3)	4 (1–9)	–	0.179^d^	29.5 (5.0)	–	0.589^e^
Group 2	65 (63.7)	2 (0–7)	–	–	29.0 (5.4)	–	–
Gender, *n* (%)							
Male	25 (24.5)	2 (0–4)	–	0.108^d^	29.6 (5.4)	–	0.385^e^
Female	77 (75.5)	4 (0–9)	–	–	28.9 (5.2)	–	–
Marriage, *n* (%)							
Single	56 (54.9)	3 (0–7)	–	0.345^d^	29.0 (5.2)	–	0.811^e^
Married	46 (45.1)	3 (1–9)	–	–	29.3 (5.3)	–	–
Occupation, *n* (%)							
Doctor	40 (39.2)	3 (0–7)	–	0.456^d^	28.9 (4.8)	–	0.384^e^
Nurse	54 (52.9)	4 (0–9)	–	–	29.0 (5.3)	–	–
Technician	8 (7.8)	2 (0–5)	–	–	31.6 (7.1)	–	–
Contact with COVID-19 patients or specimens, *n* (%)							
Yes	93 (91.2)	3 (0–8)	–	1.000^d^	28.9 (5.2)	–	0.134^e^
No	9 (8.8)	2 (0–8)	–	–	31.7 (4.7)	–	–

IQR, interquartile range.

a. Spearman correlation coefficient rho between sociodemographic data and IES-R.

b. Spearman correlation coefficient rho between sociodemographic data and GSES.

c. *P-*value for Spearman correlation coefficient.

d. *P-*value for Mann–Whitney *U*-test of IES-R scores in different groups.

e. *P-*value for independent-sample *t*-test of GSES scores in different groups.

### The Sources of distress score and the IES-R score

The health of one's family/others, the virus spread, changes in work and one's own health were the top four sources of distress. The total score of sources of distress was moderately correlated with the IES-R score (rho = 0.501, *P*<0.001) and was higher in group 1 of healthcare staff compared with healthcare staff in group 2 (Table 3). The source of distress score had a weak correlation with the GSES score, and negatively affected the GSES score (rho = −0.239, *P* = 0.016)

**Table 3:** The sources of distress among medical workers at the fever clinic and their correlation with Impact of Event Scale-Revised (IES-R) scores and the General Self-Efficacy Scale (GSES) scores

	Median (IQR) (*n* = 102)	*P*^a^	IES-R^b^ (*n* = 100)	*P*^c^	GSES^b^ (*n* = 102)	*P*^c^
One's own health	0.25 (0.25–0.75)		0.390	<0.001	−0.171	0.087
Group 1	0.50 (0.25–1.12)	0.046	0.416	0.013	−0.273	0.103
Group 2	0.25 (0.25–0.75)	–	0.329	0.007	−0.155	0.218
Health of one's family/others	0.88 (0.25–1.25)		0.365	<0.001	−0.166	0.096
Group 1	1.00 (0.38–1.50)	0.380	0.339	0.053	−0.136	0.424
Group 2	0.75 (0.25–1.25)	–	0.367	0.003	−0.201	0.108
Virus spread	0.50 (0.00–1.00)		0.420	<0.001	−0.099	0.321
Group 1	1.00 (0.50–1.00)	0.014	0.396	0.018	−0.239	0.155
Group 2	0.50 (0.00–1.00)	–	0.404	0.001	−0.072	0.571
Vulnerability/loss of control	0.00 (0.00–0.75)		0.384	<0.001	−0.206	0.037
Group 1	0.25 (0.00–0.75)	0.012	0.446	0.007	−0.275	0.099
Group 2	0.00 (0.00–0.50)	–	0.325	0.008	−0.204	0.103
Changes in work	0.50 (0.00–1.00)		0.381	<0.001	−0.196	0.048
Group 1	0.50 (0.00–1.00)	0.206	0.333	0.050	−0.129	0.447
Group 2	0.50 (0.00–1.00)	–	0.382	0.002	−0.267	0.032
Being isolated	0.00 (0.00–0.67)		0.280	0.005	−0.171	0.085
Group 1	0.00 (0.00–0.67)	0.376	0.166	0.339	−0.183	0.279
Group 2	0.00 (0.00–0.67)	–	0.326	0.008	−0.188	0.133
Total score	0.44 (0.22–0.94)		0.501	<0.001	−0.239	0.016
Group 1	0.50 (0.30–1.00)	0.019	0.510	0.002	−0.279	0.095
Group 2	0.33 (0.17–0.78)	–	0.459	<0.001	−0.272	0.028

IQR, interquartile range.

a. *P*-value for Mann–Whitney *U*-test to compare the scores of the Source of Distress between the first batch and the second batch of medical workers.

b. Spearman correlation coefficient rho between scores of the Sources of Distress and IES-R/GSES.

c. *P*-value for Spearman correlation analysis.

## Discussion

### Findings from SARS

In the circumstances of the COVID-19 pandemic, front-line healthcare staff are caring for patients in this emergent situation with a shortage of human resources and facilities. During the outbreak of SARS, which was regarded as an acute episode of a bio-disaster in 2003, several studies focused on the psychological impact of SARS on healthcare staff in different regions. In a tertiary hospital in Taiwan, the estimated prevalence of psychiatric morbidity in healthcare staff measured by the Chinese Health Questionnaire was 75.3%.^18^ In a study conducted in Toronto, 29% of healthcare staff in a tertiary hospital scored above the threshold on the General Health Questionnaire (GHQ-12), indicating probable emotional distress.^19^ A high degree of distress, indicated by a high score on the IES, was experienced by 17.7–36.0% of hospital workers in Canada, Hong Kong, Singapore and Beijing.^4–7^

### Findings from other studies during the COVID-19 outbreak

During the COVID-19 outbreak in China, 36% of healthcare staff experienced moderate-to-severe distress symptoms (IES-R≥26), as reported by Lai and colleagues.^19^ Participants reported experiencing psychological burden, especially nurses, women, people in Wuhan, and front-line healthcare workers directly engaged in the diagnosis, treatment and care of patients with COVID-19.^20^

### Our main findings and comparison with findings from other studies

Healthcare staff in the current study were under great stress, consistent with other similar situations. However, the proportion of abnormal IES-R scores was 6.0%, which was significantly lower than that of previous studies during SARS^4–7^ and other findings among healthcare staff during the COVID-19 outbreak in China,^20^ suggesting that the stress level indicated by IES-R scores was not elevated in the current study. As reported by Lai and colleagues, 27% of healthcare staff (including both front-line and second-line healthcare staff) experienced moderate-to-severe distress symptoms (IES-R≥26) outside Hubei Province, and the median for IES-R was 15.0 (IQR = 4.0–26.0).^20^ Beijing is one of the highest incidence regions for the epidemic of COVID-19 outside Hubei Province,^21^ and the significantly lower stress level in the current study could not be explained by the less severe epidemic of COVID-19 in Beijing. It is assumed that the stress management procedures may buffer the negative impact of stress.

For medical professionals who work with an acute infectious disease that is highly contagious and has a high mortality rate, working at fever clinics is challenging. The psychosocial effects of extreme events have commonly been viewed as resulting from a complex array of primary and secondary stressors.^22,23^ Primary stressors are the sources of worry, anxiety or stress that stem directly from the events and consequential tasks that the staff of services face. Secondary stressors are matters present prior to an emergency, incident or disaster or that arise during events or subsequently.

Healthcare staff during an outbreak of SARS reported both primary and secondary stressors, including that their job put them at higher risk, more stress at work, greater concerns about their own health, a greater tendency to fear discrimination and greater fear of passing on SARS to their family, which may be associated with post-traumatic stress levels.^5,14,24^ In our study, worries about the health of one's family/others, one's own health, virus transmission and changes in work were the top four sources of distress for healthcare staff. Worries about one's own health and virus transmission to others were stressors directly related to the epidemic, and therefore are primary stressors. Whereas worries about the health of one's family/others were also largely due to worries about their lack of access to food and medical care, and therefore partly secondary stressors. Changes in work (schedule and load) in our study were partly caused indirectly by the epidemic as a result of lack of personnel and protective resources, and therefore partly secondary stressors.

All these four main stressors were targeted in our study including supervision of personal protection equipment wearing, strict protection procedures, psychological support, taking care of family members of front-line healthcare staff, and reasonable adjustments of working hours according to feedback.

Changes in their working conditions, such as adjustment to their work hours, shift patterns and providing adequate protective clothing, were made during the time period of this study. It therefore does affect the consistency of the data, especially for the healthcare staff in the first group. As a result, the IES-R scores in the first group were a little bit higher than in the second group (*P* = 0.179).

Low internal consistency reliability (Cronbach's alpha of 0.483 and 0.367) resulted for the two subscales ‘virus spread’ and ‘isolation’ in the sources of distress scale, which significantly limits their validity. Consequently, the authors advise caution when interpreting the data from these two subscales. Considering the good internal consistency reliability of the scale (Cronbach's alpha = 0.886), the total score for the sources of distress measure may be more valid.

The average score on GSES of health workers in this study was 29.5 (s.d. = 5.4), higher than that of other studies in China.^25,26^ Many factors affect the self-efficacy of healthcare staff, such as the selection criteria for the assignment of the healthcare staff to the fever clinic in the current study, team dynamics, vicarious experience, verbal persuasion from others and training in doctor–patient communication skills.^27–29^ Supervision of protection procedures was available to every medical worker in the study. Demonstration and approbation from supervisors, working as a team member rather than alone, may have a positive effect on self-efficacy. A training course on doctor–patient communication skills, as well as Balint group work, has been available, since 2009, to undergraduates, postgraduates, residents and nurses in the hospital enrolled in the study.^30,31^ Self-efficacy affects an individual's assessment of and style of coping with stress.^32^ There is a correlation between the GSES score and the source of distress score, which is weakly negative, consistent with the results mentioned above.

### Limitations

The study has several limitations, including the single-centre design, which limits the generalizability of the study. The courage and professionalism of healthcare staff during the COVID-19 pandemic did not make them immune to stress. In spite of the generalizability of the study, it is worthwhile to implement the stress management procedure in frontline healthcare staff. The second limitation was the limited sample size compared with other cross-sectional studies focused on the psychological impact of the COVID-19 outbreak on healthcare staff.^20,33^ Qualitative and quantitative (including self-administered questionnaires) interviews were conducted via telephone in the current study. This process was time consuming but gave hotline workers the opportunity to talk with front-line healthcare staff, understand their feelings and needs, and provide necessary support. Given their extensive work in such an emergent situation with a shortage of resources, they need and deserve support from their colleagues (such as the support supplied by the hotline). The third limitation was the lack of a comparison group without stress management procedures to verify if there was a difference in the sources of distress and IES-R scores in the two groups.

### Implications

In conclusion, during the COVID-19 pandemic, the stress levels of healthcare staff in the fever clinic of a tertiary general hospital in Beijing were not elevated. It is rational that physio-psychosocial interventions, including the fulfilment of basic needs, the activation of self-efficacy and psychological support, may buffer the negative impact of such an event and are worth recommending in fighting COVID-19.

## Data Availability

The data that support the findings of this study are available from the corresponding author, J.W., upon reasonable request.
